# Correction: Maintenance of divergent lineages of the Rice Blast Fungus *Pyricularia oryzae* through niche separation, loss of sex and post-mating genetic incompatibilities

**DOI:** 10.1371/journal.ppat.1010944

**Published:** 2022-11-07

**Authors:** Maud Thierry, Florian Charriat, Joëlle Milazzo, Henri Adreit, Sébastien Ravel, Sandrine Cros-Arteil, Sonia borron, Violaine Sella, Thomas Kroj, Renaud Ioos, Elisabeth Fournier, Didier Tharreau, Pierre Gladieux

S3 Table lists short read sequence data of six isolates as provided on request. The authors have provided the accession numbers for these reads in the updated S3 Table.

## Supporting information

S3 Table Sequenced isolates. (XLSX)
