# Supplementary material for: Maintenance of divergent lineages of the Rice Blast Fungus Pyricularia oryzae through niche separation, loss of sex and post-mating genetic incompatibilities
Source: PLoS Pathog. 2022 Jul 25;18(7):e1010687. doi: 10.1371/journal.ppat.1010687 (PMC9352207; doi:10.1371/journal.ppat.1010687)
Supplement: S1 Text — (DOCX) [file ppat.1010687.s019.docx]

**S1 Text.**

1. F_ST_ between lineages, computed from Infinium SNPs, or SNPs identified in whole genome sequencing data

We present F_ST_ estimated from Infinium data (S1 Table) between lineages (Table A) and between clusters within lineage 1 (Table B), and F_ST_ estimated from this study’s whole genome sequencing data (S3 Table) between lineages (Table C). We also present inter-lineage F_ST_ estimated from the whole genome sequences used to design our Infinium genotyping beadchip (Table D), i.e. 25 paired-end Illumina genomes from (1) and four genomes of rice-infecting isolates from (2). All calculations were carried out using Egglib v3. For F_ST_ estimated from whole genome sequencing data, sites with more than 30% missing data were excluded (no missing data in our Infinium dataset).

Inter-lineage F_ST_ estimated from Infinium data was consistently higher than inter-lineage F_ST_ estimated from SNPs identified using this study’s whole genome sequencing data (Table A; Table C). However, inter-lineage F_ST_ estimated from Infinium data was not markedly different from inter-lineage F_ST_ estimated the whole genome sequencing data used to design the Infinium beadchip, which indicates that differences in F_ST_ between Infinium data and this study’s whole genome sequencing data was caused by differences in sample sets and not by an ascertainment bias in measurements of population differentiation. The lack of ascertainment bias is consistent with the fact that Infinium SNPs were selected to be biallelic in the whole dataset, and not to discriminate the different lineages (see Methods).

1. F_ST_ between lineages, computed for effector and non-effector genes

Mann-Whitney U-tests were performed to compare inter-lineage F_ST_ in effector genes and non-effector genes. None of the comparisons were statistically significant (p-value > 0.05). Box plots of inter-lineage F_ST_ are presented in Fig A.

Table A. Inter-lineage F_ST_ estimated from Infinium data (n=886 isolates)

| Lineages | 1 | 2 | 3 |
| --- | --- | --- | --- |
| 2 | 0.688 |  |  |
| 3 | 0.732 | 0.940 |  |
| 4 | 0.565 | 0.913 | 0.932 |

Table B. Inter-cluster F_ST_ estimated from Infinium data (n=189 isolates)

| Clusters in lineage 1 | International | Laos | Baoshan |
| --- | --- | --- | --- |
| Laos | 0.209 |  |  |
| Baoshan | 0.337 | 0.415 |  |
| Yule | 0.272 | 0.203 | 0.506 |

Table C. Inter-lineage F_ST_ estimated from this study’s whole genome sequencing data (n=123 isolates)

| Lineages | 1 | 2 | 3 |
| --- | --- | --- | --- |
| 2 | 0.358 |  |  |
| 3 | 0.350 | 0.641 |  |
| 4 | 0.243 | 0.634 | 0.615 |

Table D. Inter-lineage F_ST_ estimated from whole genome sequencing data used to design the Infinium beadchip (25 genomes from (1); four genomes from (2)).

| Lineages | 1 | 2 | 3 |
| --- | --- | --- | --- |
| 2 | 0.710 |  |  |
| 3 | 0.729 | 0.867 |  |
| 4 | 0.587 | 0.848 | 0.851 |


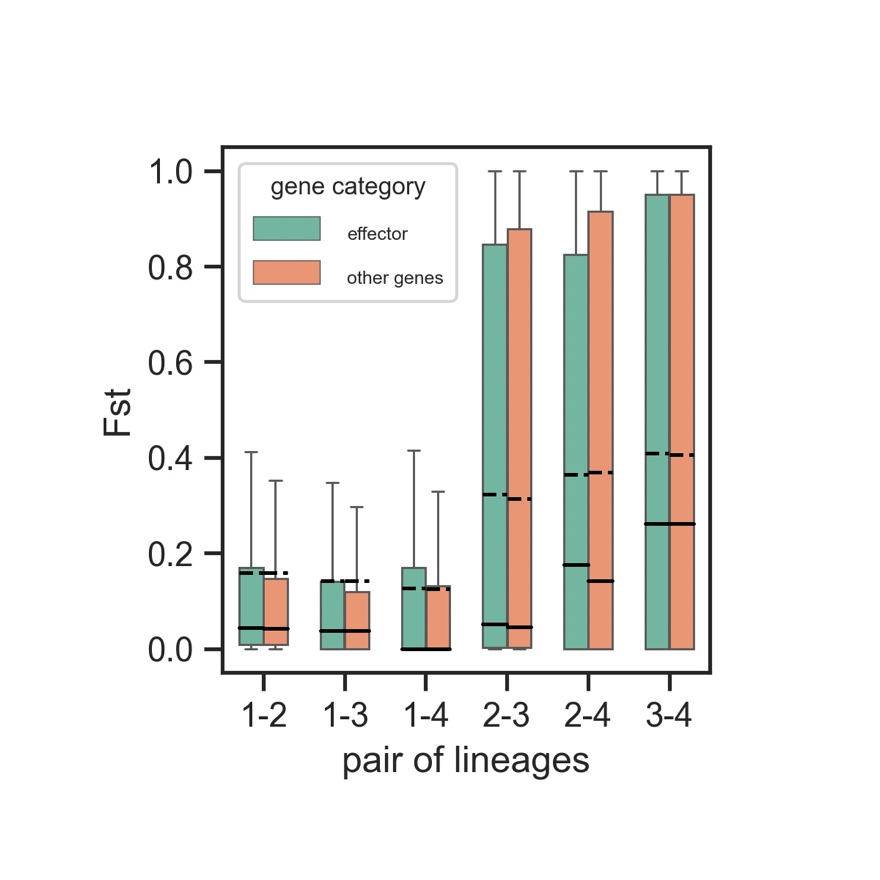


Fig A. Inter-lineage F_ST_ at effector genes and other genes. Dashed and solid black lines represent the mean and median, respectively.

**References**

1. Gladieux P, Ravel S, Rieux A, Cros-Arteil S, Adreit H, Milazzo J, et al. Coexistence of multiple endemic and pandemic lineages of the rice blast pathogen. mBio. 2018;9(2).

2. Chiapello H, Mallet L, Guerin C, Aguileta G, Amselem J, Kroj T, et al. Deciphering Genome Content and Evolutionary Relationships of Isolates from the Fungus Magnaporthe oryzae Attacking Different Host Plants. Genome Biol Evol. 2015;7(10):2896-912.
