# Supplementary material for: Maintenance of divergent lineages of the Rice Blast Fungus Pyricularia oryzae through niche separation, loss of sex and post-mating genetic incompatibilities
Source: PLoS Pathog. 2022 Jul 25;18(7):e1010687. doi: 10.1371/journal.ppat.1010687 (PMC9352207; doi:10.1371/journal.ppat.1010687)
Supplement: S6 Text — (DOCX) [file ppat.1010687.s024.docx]

S6 Text: Analysis of mycelial growth rates

We measured growth rate of 41 representative isolates cultured at different temperatures to test the hypothesis of adaptation to temperature. Main results are shown in Fig A.


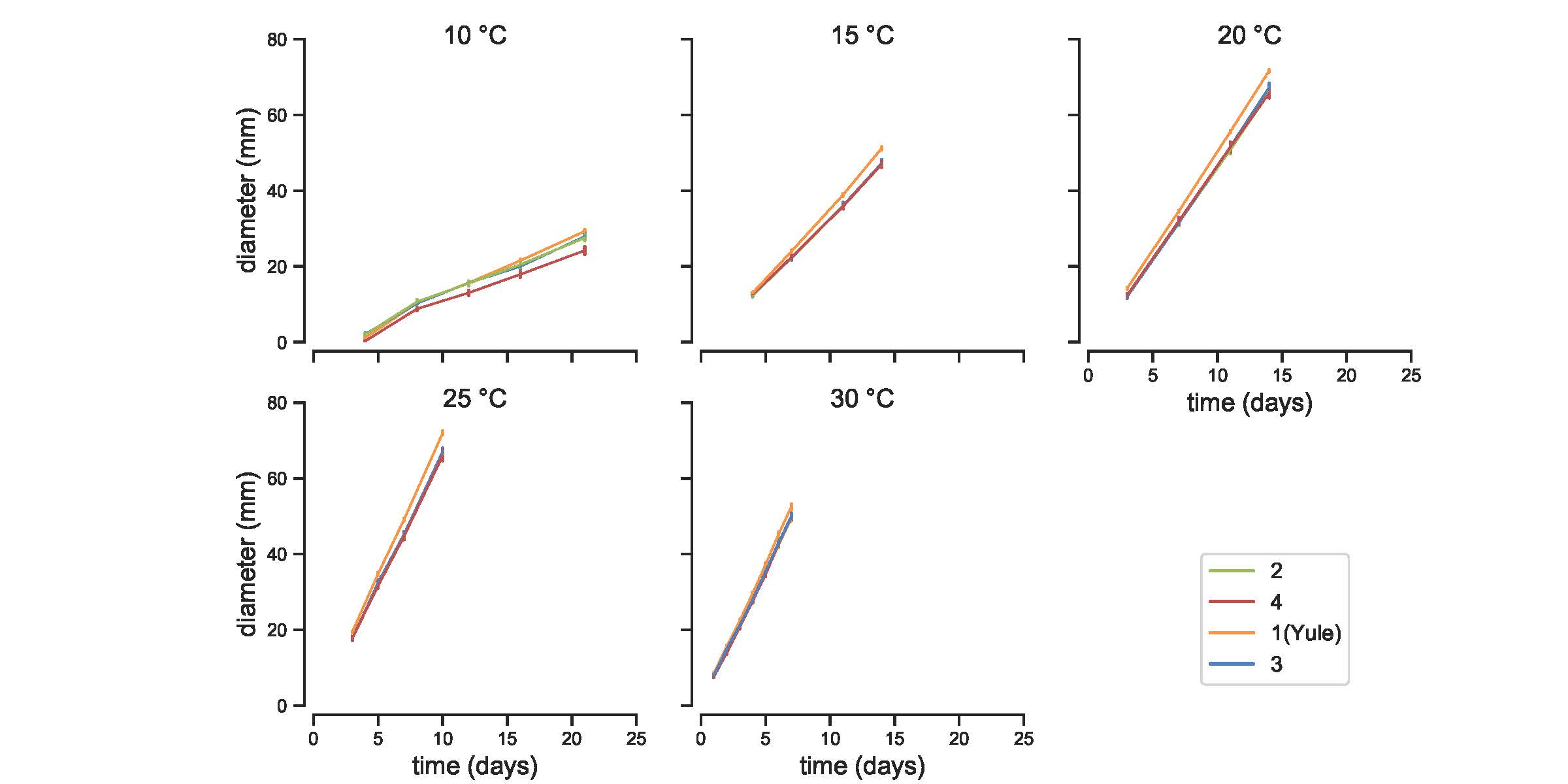


Fig A. Growth curves for lineages 2-4 and cluster *Yule* within lineage 1 of *P. oryzae* at five incubation temperatures. Error bars represent the standard error across three to four biological replicates.

In what follows, we detail the statistical processing of data:

#R packages (R version 4.0.3)
library(lme4)

library(ggplot2)

library(lmerTest)

library(multcomp)

library(lsmeans)

library(RVAideMemoire)

#load and process data
temp_data <- read.table("S11 Data.txt", sep="\t",header=T)
temp_data$Temperature<-as.factor(temp_data$Temperature)
temp_data$isolate<-as.factor(temp_data$isolate)
temp_data$lineage<-as.factor(temp_data$lineage)
temp_data$replicate<-as.factor(temp_data$replicate)

#T=10°C data summary
T10<- temp_data[ which(temp_data$Temperature==10 & temp_data$diameter>0), ]
T10<-droplevels(T10)

#T=10°C plot
ggplot(T10, aes( time,diameter, color=lineage)) +
 stat_summary(fun.data=mean_se, geom="pointrange") + scale_color_manual(values=c("#FFD479", "#9BBB59", "#4F81BD","#C0504D"))

#T=10°C: linear mixed model
m.10 <- lmer(sqrt(diameter) ~ time + replicate+ lineage +(1 | isolate), data=T10, REML=T)

#T=10°C: plot residuals

plotresid(m.10)

## Registered S3 methods overwritten by 'car':
## method from
## influence.merMod lme4
## cooks.distance.influence.merMod lme4
## dfbeta.influence.merMod lme4
## dfbetas.influence.merMod lme4

#T=10°C: ANOVA

anova( m.10)

## Type III Analysis of Variance Table with Satterthwaite's method
## Sum Sq Mean Sq NumDF DenDF F value Pr(>F)
## time 276.203 276.203 1 364.39 7987.5139 < 2.2e-16 ***
## replicate 3.317 1.658 2 367.46 47.9561 < 2.2e-16 ***
## lineage 0.528 0.176 3 35.21 5.0867 0.004972 **
## ---
## Signif. codes: 0 '***' 0.001 '**' 0.01 '*' 0.05 '.' 0.1 ' ' 1

summary(m.10)

## Linear mixed model fit by REML. t-tests use Satterthwaite's method [
## lmerModLmerTest]
## Formula: sqrt(diameter) ~ time + replicate + lineage +
## (1 | isolate)
## Data: T10
##
## REML criterion at convergence: -79.3
##
## Scaled residuals:
## Min 1Q Median 3Q Max
## -3.7622 -0.6272 0.0342 0.6412 2.1850
##
## Random effects:
## Groups Name Variance Std.Dev.
## isolate (Intercept) 0.04725 0.2174
## Residual 0.03458 0.1860
## Number of obs: 406, groups: isolate, 39
##
## Fixed effects:
## Estimate Std. Error df t value Pr(>|t|)
## (Intercept) 2.079740 0.073437 48.001647 28.320 < 2e-16 ***
## time 0.158814 0.001777 364.390128 89.373 < 2e-16 ***
## replicate2 -0.166588 0.021601 365.873700 -7.712 1.19e-13 ***
## replicate3 0.049812 0.025184 368.435269 1.978 0.048685 *
## lineage2 -0.056168 0.101040 34.939604 -0.556 0.581824
## lineage3 -0.063920 0.101069 34.978664 -0.632 0.531213
## lineage4 -0.356141 0.098702 35.598575 -3.608 0.000939 ***
## ---
## Signif. codes: 0 '***' 0.001 '**' 0.01 '*' 0.05 '.' 0.1 ' ' 1
##
## Correlation of Fixed Effects:
## (Intr) tmps__ rpttn2 rpttn3 ligne2 ligne3
## tmps_d_cltr -0.344
## replicate2 -0.150 -0.003
## replicate3 -0.186 0.166 0.445
## lineage2 -0.621 0.003 0.002 -0.004
## lineage3 -0.619 0.002 -0.004 -0.016 0.451
## lineage4 -0.634 -0.003 -0.008 0.013 0.461 0.461

#T=10°C: model fit
ggplot(T10, aes( time,sqrt(diameter), color=lineage)) +
 stat_summary(fun.data=mean_se, geom="pointrange")+
 stat_summary(aes(y=fitted(m.10)),fun=mean,geom="line")

## Warning: Removed 1 rows containing missing values (geom_segment).

#T=10°C : least-squares means
LSM<-lsmeans(m.10,pairwise~lineage)

LSM

## $lsmeans
## lineage lsmean SE df lower.CL upper.CL
## 1(Yule) 4.20 0.0679 34.9 4.06 4.34
## 2 4.15 0.0749 34.6 3.99 4.30
## 3 4.14 0.0749 34.6 3.99 4.29
## 4 3.85 0.0718 36.0 3.70 3.99
##
## Results are averaged over the levels of: replicate
## Degrees-of-freedom method: kenward-roger
## Results are given on the sqrt (not the response) scale.
## Confidence level used: 0.95
##
## $contrasts
## contrast estimate SE df t.ratio p.value
## 1(Yule) - 2 0.05617 0.1010 34.7 0.556 0.9443
## 1(Yule) - 3 0.06392 0.1011 34.8 0.632 0.9209
## 1(Yule) - 4 0.35614 0.0987 35.4 3.608 0.0050
## 2 - 3 0.00775 0.1059 34.6 0.073 0.9999
## 2 - 4 0.29997 0.1037 35.2 2.894 0.0315
## 3 - 4 0.29222 0.1037 35.2 2.818 0.0377
##
## Results are averaged over the levels of: replicate
## Note: contrasts are still on the sqrt scale
## Degrees-of-freedom method: kenward-roger
## P value adjustment: tukey method for comparing a family of 4 estimates

cld(lsmeans(m.10,~lineage))

## Note: Use 'contrast(regrid(object), ...)' to obtain contrasts of back-transformed estimates

## lineage lsmean SE df lower.CL upper.CL .group
## 4 3.85 0.0718 36.0 3.70 3.99 1
## 3 4.14 0.0749 34.6 3.99 4.29 2
## 2 4.15 0.0749 34.6 3.99 4.30 2
## 1(Yule) 4.20 0.0679 34.9 4.06 4.34 2
##
## Results are averaged over the levels of: replicate
## Degrees-of-freedom method: kenward-roger
## Results are given on the sqrt (not the response) scale.
## Confidence level used: 0.95
## Note: contrasts are still on the sqrt scale
## P value adjustment: tukey method for comparing a family of 4 estimates
## significance level used: alpha = 0.05

#T=15°C data summary
T15<- temp_data[ which(temp_data$Temperature==15 & temp_data$diameter>0), ]
T15<-droplevels(T15)

#T=15°C plot
ggplot(T15, aes( time,diameter, color=lineage)) +
 stat_summary(fun.data=mean_se, geom="pointrange") *+ scale_color_manual(values=c("#FFD479", "#9BBB59", "#4F81BD","#C0504D"))*

#T=15°C: linear mixed model
m.15 <- lmer(diameter ~ time + replicate+ lineage +(1 | isolate), data=T15, REML=T)

#T=15°C: plot residuals
plotresid(m.15)

#T=15°C: ANOVA

anova( m.15)

## Type III Analysis of Variance Table with Satterthwaite's method
## Sum Sq Mean Sq NumDF DenDF F value Pr(>F)
## time 84476 84476 1 418.95 33161.3730 < 2.2e-16 ***
## replicate 117 59 2 420.06 23.0190 3.265e-10 ***
## lineage 35 12 3 35.90 4.6039 0.007944 **
## ---
## Signif. codes: 0 '***' 0.001 '**' 0.01 '*' 0.05 '.' 0.1 ' ' 1

summary(m.15)

## Linear mixed model fit by REML. t-tests use Satterthwaite's method [
## lmerModLmerTest]
## Formula: diameter ~ time + replicate + lineage + (1 |
## isolate)
## Data: T15
##
## REML criterion at convergence: 1848.4
##
## Scaled residuals:
## Min 1Q Median 3Q Max
## -3.3730 -0.5755 0.0075 0.6065 3.0952
##
## Random effects:
## Groups Name Variance Std.Dev.
## isolate (Intercept) 2.898 1.702
## Residual 2.547 1.596
## Number of obs: 462, groups: isolate, 40
##
## Fixed effects:
## Estimate Std. Error df t value Pr(>|t|)
## (Intercept) 0.35650 0.56983 46.78738 0.626 0.53460
## time 3.54757 0.01948 418.95477 182.103 < 2e-16 ***
## replicate2 -1.20323 0.18088 419.58573 -6.652 9.04e-11 ***
## replicate3 -0.37797 0.18394 420.47805 -2.055 0.04051 *
## lineage2 -2.46584 0.79717 36.32295 -3.093 0.00380 **
## lineage3 -2.20758 0.77054 35.53873 -2.865 0.00696 **
## lineage4 -2.28674 0.77054 35.53873 -2.968 0.00534 **
## ---
## Signif. codes: 0 '***' 0.001 '**' 0.01 '*' 0.05 '.' 0.1 ' ' 1
##
## Correlation of Fixed Effects:
## (Intr) tmps__ rpttn2 rpttn3 ligne2 ligne3
## tmps_d_cltr -0.309
## replicate2 -0.161 0.010
## replicate3 -0.160 0.002 0.485
## lineage2 -0.627 0.004 0.011 0.022
## lineage3 -0.644 0.000 0.000 0.000 0.460
## lineage4 -0.644 0.000 0.000 0.000 0.460 0.476

#T=15°C: model fit
ggplot(T15, aes( time,diameter, color=lineage)) +
 stat_summary(fun.data=mean_se, geom="pointrange")+
 stat_summary(aes(y=fitted(m.15)),fun=mean,geom="line")

#T=15°C: least-squares means
LSM<-lsmeans(m.15,pairwise~lineage)
LSM

## $lsmeans
## lineage lsmean SE df lower.CL upper.CL
## 1(Yule) 31.7 0.532 35.7 30.6 32.8
## 2 29.2 0.594 37.1 28.0 30.4
## 3 29.5 0.558 35.7 28.3 30.6
## 4 29.4 0.558 35.7 28.3 30.5
##
## Results are averaged over the levels of: replicate
## Degrees-of-freedom method: kenward-roger
## Confidence level used: 0.95
##
## $contrasts
## contrast estimate SE df t.ratio p.value
## 1(Yule) - 2 2.4658 0.797 36.5 3.093 0.0190
## 1(Yule) - 3 2.2076 0.771 35.7 2.865 0.0335
## 1(Yule) - 4 2.2867 0.771 35.7 2.968 0.0262
## 2 - 3 -0.2583 0.815 36.4 -0.317 0.9888
## 2 - 4 -0.1791 0.815 36.4 -0.220 0.9962
## 3 - 4 0.0792 0.789 35.7 0.100 0.9996
##
## Results are averaged over the levels of: replicate
## Degrees-of-freedom method: kenward-roger
## P value adjustment: tukey method for comparing a family of 4 estimates

cld(lsmeans(m.15,~lineage))

## lineage lsmean SE df lower.CL upper.CL .group
## 2 29.2 0.594 37.1 28.0 30.4 1
## 4 29.4 0.558 35.7 28.3 30.5 1
## 3 29.5 0.558 35.7 28.3 30.6 1
## 1(Yule) 31.7 0.532 35.7 30.6 32.8 2
##
## Results are averaged over the levels of: replicate
## Degrees-of-freedom method: kenward-roger
## Confidence level used: 0.95
## P value adjustment: tukey method for comparing a family of 4 estimates
## significance level used: alpha = 0.05

#T=20°C data summary
T20<- temp_data[ which(temp_data$Temperature==20 & temp_data$diameter>0), ]
T20<-droplevels(T20)

#T=20°C plot
ggplot(T20, aes( time,diameter, color=lineage)) +
 stat_summary(fun.data=mean_se, geom="pointrange") *+ scale_color_manual(values=c("#FFD479", "#9BBB59", "#4F81BD","#C0504D"))*

#T=20°C: linear mixed model
m.20 <- lmer(diameter ~ time + replicate+ lineage +(1 | isolate), data=T20, REML=T)

#T=20°C: plot residuals
plotresid(m.20)

#T=20°C: ANOVA

anova( m.20)

## Type III Analysis of Variance Table with Satterthwaite's method
## Sum Sq Mean Sq NumDF DenDF F value Pr(>F)
## time 261525 261525 1 542.24 40334.8449 < 2.2e-16 ***
## replicate 262 87 3 544.73 13.4525 1.763e-08 ***
## lineage 131 44 3 37.42 6.7385 0.0009527 ***
## ---
## Signif. codes: 0 '***' 0.001 '**' 0.01 '*' 0.05 '.' 0.1 ' ' 1

summary(m.20)

## Linear mixed model fit by REML. t-tests use Satterthwaite's method [
## lmerModLmerTest]
## Formula: diameter ~ time + replicate + lineage + (1 |
## isolate)
## Data: T20
##
## REML criterion at convergence: 2852.5
##
## Scaled residuals:
## Min 1Q Median 3Q Max
## -3.7249 -0.6031 -0.0231 0.5991 3.7700
##
## Random effects:
## Groups Name Variance Std.Dev.
## isolate (Intercept) 4.690 2.166
## Residual 6.484 2.546
## Number of obs: 586, groups: isolate, 41
##
## Fixed effects:
## Estimate Std. Error df t value Pr(>|t|)
## (Intercept) -0.02756 0.72532 46.84460 -0.038 0.969846
## time 4.95997 0.02470 542.23625 200.835 < 2e-16 ***
## replicate2 -0.10044 0.29087 543.55006 -0.345 0.730001
## replicate3 1.01102 0.32489 545.15609 3.112 0.001956 **
## replicate4 1.46067 0.28775 546.62482 5.076 5.29e-07 ***
## lineage2 -3.73244 0.98908 36.80540 -3.774 0.000567 ***
## lineage3 -3.51584 0.99726 37.88113 -3.525 0.001124 **
## lineage4 -3.51914 0.99135 37.14063 -3.550 0.001066 **
## ---
## Signif. codes: 0 '***' 0.001 '**' 0.01 '*' 0.05 '.' 0.1 ' ' 1
##
## Correlation of Fixed Effects:
## (Intr) tmps__ rpttn2 rpttn3 rpttn4 ligne2 ligne3
## tmps_d_cltr -0.243
## replicate2 -0.157 -0.131
## replicate3 -0.138 -0.134 0.444
## replicate4 -0.156 -0.143 0.497 0.455
## lineage2 -0.651 0.001 -0.001 -0.003 0.001
## lineage3 -0.643 0.002 -0.001 -0.010 -0.020 0.473
## lineage4 -0.650 0.006 -0.003 0.003 -0.002 0.476 0.472

#T=20°C: model fit
ggplot(T20, aes( time,diameter, color=lineage)) +
 stat_summary(fun.data=mean_se, geom="pointrange")+
 stat_summary(aes(y=fitted(m.20)),fun=mean,geom="line")

#T=20°C: least-squares means
LSM<-lsmeans(m.20,pairwise~lineage)
LSM

## $lsmeans
## lineage lsmean SE df lower.CL upper.CL
## 1(Yule) 41.5 0.683 36.6 40.1 42.8
## 2 37.7 0.715 36.3 36.3 39.2
## 3 37.9 0.727 38.3 36.5 39.4
## 4 37.9 0.719 37.0 36.5 39.4
##
## Results are averaged over the levels of: replicate
## Degrees-of-freedom method: kenward-roger
## Confidence level used: 0.95
##
## $contrasts
## contrast estimate SE df t.ratio p.value
## 1(Yule) - 2 3.73244 0.989 36.4 3.774 0.0031
## 1(Yule) - 3 3.51584 0.997 37.4 3.525 0.0060
## 1(Yule) - 4 3.51914 0.991 36.7 3.550 0.0057
## 2 - 3 -0.21660 1.020 37.3 -0.212 0.9966
## 2 - 4 -0.21329 1.014 36.6 -0.210 0.9966
## 3 - 4 0.00331 1.022 37.6 0.003 1.0000
##
## Results are averaged over the levels of: replicate
## Degrees-of-freedom method: kenward-roger
## P value adjustment: tukey method for comparing a family of 4 estimates

cld(lsmeans(m.20,~lineage))

## lineage lsmean SE df lower.CL upper.CL .group
## 2 37.7 0.715 36.3 36.3 39.2 1
## 4 37.9 0.719 37.0 36.5 39.4 1
## 3 37.9 0.727 38.3 36.5 39.4 1
## 1(Yule) 41.5 0.683 36.6 40.1 42.8 2
##
## Results are averaged over the levels of: replicate
## Degrees-of-freedom method: kenward-roger
## Confidence level used: 0.95
## P value adjustment: tukey method for comparing a family of 4 estimates
## significance level used: alpha = 0.05

#T=25°C data summary
T25<- temp_data[ which(temp_data$Temperature==25 & temp_data$diameter>0), ]
T25<-droplevels(T25)

#T=25°C plot
ggplot(T25, aes( time,diameter, color=lineage)) +
 stat_summary(fun.data=mean_se, geom="pointrange") *+ scale_color_manual(values=c("#FFD479", "#9BBB59", "#4F81BD","#C0504D"))*

#T=25°C: linear mixed model
m.25 <- lmer(diameter ~ time + replicate+ lineage +(1 | isolate), data=T25, REML=T)

#T=25°C: plot residuals
plotresid(m.25)

anova( m.25)

## Type III Analysis of Variance Table with Satterthwaite's method
## Sum Sq Mean Sq NumDF DenDF F value Pr(>F)
## time 151922 151922 1 412.75 30155.7746 < 2.2e-16 ***
## replicate 716 358 2 413.29 71.0545 < 2.2e-16 ***
## lineage 102 34 3 36.19 6.7492 0.0009877 ***
## ---
## Signif. codes: 0 '***' 0.001 '**' 0.01 '*' 0.05 '.' 0.1 ' ' 1

summary(m.25)

## Linear mixed model fit by REML. t-tests use Satterthwaite's method [
## lmerModLmerTest]
## Formula: diameter ~ time + replicate + lineage + (1 |
## isolate)
## Data: T25
##
## REML criterion at convergence: 2114.1
##
## Scaled residuals:
## Min 1Q Median 3Q Max
## -5.2596 -0.6017 0.0232 0.5864 2.5340
##
## Random effects:
## Groups Name Variance Std.Dev.
## isolate (Intercept) 3.958 1.990
## Residual 5.038 2.245
## Number of obs: 455, groups: isolate, 40
##
## Fixed effects:
## Estimate Std. Error df t value Pr(>|t|)
## (Intercept) -1.33602 0.67969 48.02757 -1.966 0.055136 .
## time 7.03059 0.04049 412.74959 173.654 < 2e-16 ***
## replicate2 0.34003 0.27474 413.62203 1.238 0.216551
## replicate3 2.77275 0.24920 413.49296 11.126 < 2e-16 ***
## lineage2 -3.20583 0.91654 36.09631 -3.498 0.001264 **
## lineage3 -3.14836 0.94356 36.19973 -3.337 0.001970 **
## lineage4 -3.59600 0.91748 36.23012 -3.919 0.000378 ***
## ---
## Signif. codes: 0 '***' 0.001 '**' 0.01 '*' 0.05 '.' 0.1 ' ' 1
##
## Correlation of Fixed Effects:
## (Intr) tmps__ rpttn2 rpttn3 ligne2 ligne3
## tmps_d_cltr -0.307
## replicate2 -0.091 -0.213
## replicate3 -0.121 -0.164 0.454
## lineage2 -0.643 -0.001 0.003 0.004
## lineage3 -0.625 -0.003 0.010 0.004 0.463
## lineage4 -0.640 -0.006 -0.002 -0.002 0.476 0.462

# model fit
ggplot(T25, aes( time,diameter, color=lineage)) +
 stat_summary(fun.data=mean_se, geom="pointrange")+
 stat_summary(aes(y=fitted(m.25)),fun=mean,geom="line")

#T=25°C: least-squares means
LSM<-lsmeans(m.25,pairwise~lineage)
LSM

## $lsmeans
## lineage lsmean SE df lower.CL upper.CL
## 1(Yule) 41.5 0.633 36.0 40.3 42.8
## 2 38.3 0.663 35.9 37.0 39.7
## 3 38.4 0.700 36.1 37.0 39.8
## 4 37.9 0.665 36.1 36.6 39.3
##
## Results are averaged over the levels of: replicate
## Degrees-of-freedom method: kenward-roger
## Confidence level used: 0.95
##
## $contrasts
## contrast estimate SE df t.ratio p.value
## 1(Yule) - 2 3.2058 0.917 35.9 3.498 0.0067
## 1(Yule) - 3 3.1484 0.944 36.0 3.337 0.0102
## 1(Yule) - 4 3.5960 0.917 36.0 3.919 0.0021
## 2 - 3 -0.0575 0.964 36.0 -0.060 0.9999
## 2 - 4 0.3902 0.939 36.0 0.416 0.9754
## 3 - 4 0.4476 0.965 36.1 0.464 0.9664
##
## Results are averaged over the levels of: replicate
## Degrees-of-freedom method: kenward-roger
## P value adjustment: tukey method for comparing a family of 4 estimates

cld(lsmeans(m.25,~lineage))

## lineage lsmean SE df lower.CL upper.CL .group
## 4 37.9 0.665 36.1 36.6 39.3 1
## 2 38.3 0.663 35.9 37.0 39.7 1
## 3 38.4 0.700 36.1 37.0 39.8 1
## 1(Yule) 41.5 0.633 36.0 40.3 42.8 2
##
## Results are averaged over the levels of: replicate
## Degrees-of-freedom method: kenward-roger
## Confidence level used: 0.95
## P value adjustment: tukey method for comparing a family of 4 estimates
## significance level used: alpha = 0.05

#T=30°C data summary
T30<- temp_data[ which(temp_data$Temperature==30 & temp_data$diameter>0), ]
T30<-droplevels(T30)

#T=30°C plot
ggplot(T30, aes( time,diameter, color=lineage)) +
 stat_summary(fun.data=mean_se, geom="pointrange") *+ scale_color_manual(values=c("#FFD479", "#9BBB59", "#4F81BD","#C0504D"))*

#T=30°C: linear mixed model
m.30 <- lmer(diameter ~ time + replicate+ lineage +(1 | isolate), data=T30, REML=T)

#T=30°C: plot residuals
plotresid(m.30)

#T=30°C: ANOVA

anova( m.30)

## Type III Analysis of Variance Table with Satterthwaite's method
## Sum Sq Mean Sq NumDF DenDF F value Pr(>F)
## time 178100 178100 1 870.34 52681.4995 < 2e-16 ***
## replicate 2063 688 3 881.49 203.4034 < 2e-16 ***
## lineage 39 13 3 37.07 3.8817 0.01649 *
## ---
## Signif. codes: 0 '***' 0.001 '**' 0.01 '*' 0.05 '.' 0.1 ' ' 1

summary(m.30)

## Linear mixed model fit by REML. t-tests use Satterthwaite's method [
## lmerModLmerTest]
## Formula: diameter ~ time + replicate + lineage + (1 |
## isolate)
## Data: T30
##
## REML criterion at convergence: 3818.9
##
## Scaled residuals:
## Min 1Q Median 3Q Max
## -3.3748 -0.5624 0.0291 0.6219 4.5766
##
## Random effects:
## Groups Name Variance Std.Dev.
## isolate (Intercept) 1.984 1.408
## Residual 3.381 1.839
## Number of obs: 915, groups: isolate, 41
##
## Fixed effects:
## Estimate Std. Error df t value Pr(>|t|)
## (Intercept) 2.48054 0.47516 48.99784 5.220 3.62e-06 ***
## time 7.19095 0.03133 870.34024 229.525 < 2e-16 ***
## replicate2 -0.14130 0.18942 872.14201 -0.746 0.45592
## replicate3 -0.08508 0.17928 885.22910 -0.475 0.63523
## replicate4 -3.54333 0.18343 886.50289 -19.317 < 2e-16 ***
## lineage2 -1.76654 0.64236 37.14510 -2.750 0.00915 **
## lineage3 -1.55366 0.63917 36.50483 -2.431 0.02010 *
## lineage4 -1.90591 0.64205 37.15585 -2.968 0.00521 **
## ---
## Signif. codes: 0 '***' 0.001 '**' 0.01 '*' 0.05 '.' 0.1 ' ' 1
##
## Correlation of Fixed Effects:
## (Intr) tmps__ rpttn2 rpttn3 rpttn4 ligne2 ligne3
## tmps_d_cltr -0.264
## replicate2 -0.195 0.003
## replicate3 -0.226 -0.002 0.511
## replicate4 -0.205 -0.070 0.499 0.604
## lineage2 -0.638 0.000 0.005 0.001 -0.007
## lineage3 -0.642 0.000 0.000 0.002 0.003 0.475
## lineage4 -0.635 0.001 0.000 -0.020 -0.018 0.473 0.475

#T=30°C: model fit
ggplot(T30, aes( time,diameter, color=lineage)) +
 stat_summary(fun.data=mean_se, geom="pointrange")+
 stat_summary(aes(y=fitted(m.30)),fun=mean,geom="line")

#T=30°C: least-squares means
LSM<-lsmeans(m.30,pairwise~lineage)
LSM

## $lsmeans
## lineage lsmean SE df lower.CL upper.CL
## 1(Yule) 31.1 0.442 36.6 30.2 32.0
## 2 29.3 0.467 37.6 28.4 30.3
## 3 29.5 0.462 36.3 28.6 30.5
## 4 29.2 0.467 37.7 28.2 30.1
##
## Results are averaged over the levels of: replicate
## Degrees-of-freedom method: kenward-roger
## Confidence level used: 0.95
##
## $contrasts
## contrast estimate SE df t.ratio p.value
## 1(Yule) - 2 1.767 0.642 37.0 2.750 0.0435
## 1(Yule) - 3 1.554 0.639 36.4 2.431 0.0891
## 1(Yule) - 4 1.906 0.642 37.0 2.968 0.0257
## 2 - 3 -0.213 0.657 36.9 -0.324 0.9880
## 2 - 4 0.139 0.660 37.5 0.211 0.9966
## 3 - 4 0.352 0.657 36.9 0.536 0.9496
##
## Results are averaged over the levels of: replicate
## Degrees-of-freedom method: kenward-roger
## P value adjustment: tukey method for comparing a family of 4 estimates

cld(lsmeans(m.30,~lineage))

## lineage lsmean SE df lower.CL upper.CL .group
## 4 29.2 0.467 37.7 28.2 30.1 1
## 2 29.3 0.467 37.6 28.4 30.3 1
## 3 29.5 0.462 36.3 28.6 30.5 12
## 1(Yule) 31.1 0.442 36.6 30.2 32.0 2
##
## Results are averaged over the levels of: replicate
## Degrees-of-freedom method: kenward-roger
## Confidence level used: 0.95
## P value adjustment: tukey method for comparing a family of 4 estimates
## significance level used: alpha = 0.05
