# Supplementary material for: Maintenance of divergent lineages of the Rice Blast Fungus Pyricularia oryzae through niche separation, loss of sex and post-mating genetic incompatibilities
Source: PLoS Pathog. 2022 Jul 25;18(7):e1010687. doi: 10.1371/journal.ppat.1010687 (PMC9352207; doi:10.1371/journal.ppat.1010687)
Supplement: S7 Text — (DOCX) [file ppat.1010687.s025.docx]

S7 Text: Analysis of sporulation data

We measured the number of spores and mycelium colony size of 41 representative isolates cultured at different temperatures to test the hypothesis of adaptation to temperature. Main results are shown in Fig A.

Fig A. Sporulation rates for lineages 2-4 and cluster *Yule* within lineage 1 of *P. oryzae* at five incubation temperatures, with three or four biological replicates. Each dot represents the median across three to four replicates. Shared superscripts indicate non-significant differences (Dunn’s non-parametric multiple comparison test, carried out only for temperatures for which Kruskal-Wallis tests were statistically significant, i.e. 10°C, 15°C and 30°C). Solid and dashed black horizontal lines represent the median and mean, respectively.

In what follows, we detail the statistical processing of data.

S7.1. Analysis of sporulation data (without weighting by mycelium size)

#R packages (R version 4.0.3)
library(ggplot2)

library(MASS)

library(ggpubr)

library(RVAideMemoire)

# library(rstatix) #import data

data_spore <- **read.csv**("S12 Data.txt", sep="\t", na.strings="na")
data_spore**$**replicate <- **as.factor**(data_spore**$**replicate)

data_spore**$**lineage <- **as.factor**(data_spore**$**lineage)

data_spore**$**isolate <- **as.factor**(data_spore**$**isolate)


summary(data_spore)

isolate temperature replicate spores lineage

CH1120 : 17 Min. :10.00 1:132 Min. : 0.0 1(Yule):155

US0032 : 17 1st Qu.:15.00 2:168 1st Qu.: 2.0 2 :124

CH0999 : 16 Median :20.00 3:165 Median : 31.0 3 :131

CH1065 : 16 Mean :20.36 4: 74 Mean : 189.2 4 :129

CL0026 : 16 3rd Qu.:27.50 3rd Qu.: 222.5

IN0072 : 16 Max. :30.00 Max. :2160.0

(Other):441

#We take the median of the number of spores across replicates
data_spore_med_rep <- aggregate(data_spore$spores,data_spore[,c("temperature","lineage","isolate")], FUN=median)
colnames(data_spore_med_rep)[4] <- 'nb_spore'

#Round medians

data_spore_med_rep**$**nb_spore <- **round**(data_spore_med_rep**$**nb_spore)

#Make one dataset per temperature

data_sp_10_med <- **subset**(data_spore_med_rep, temperature**==**10)

data_sp_15_med <- **subset**(data_spore_med_rep, temperature**==**15)

data_sp_20_med <- **subset**(data_spore_med_rep, temperature**==**20)

data_sp_25_med <- **subset**(data_spore_med_rep, temperature**==**25)

data_sp_30_med <- **subset**(data_spore_med_rep, temperature**==**30)

#T=10°C: plot

ggplot(data_sp_10_med, aes( lineage,nb_spore, color=lineage))+ geom_boxplot()+ facet_wrap(~ temperature) + scale_color_manual(values=c("#FFD479", "#9BBB59", "#4F81BD","#C0504D"))+ labs(y="Number of spores", x="Lineages")

#T=15°C: plot

ggplot(data_sp_15_med, aes( lineage,nb_spore, color=lineage))+ geom_boxplot()+ facet_wrap(~ temperature) + scale_color_manual(values=c("#FFD479", "#9BBB59", "#4F81BD","#C0504D"))+ labs(y="Number of spores", x="Lineages")

#T=20°C: plot

ggplot(data_sp_20_med, aes( lineage,nb_spore, color=lineage))+ geom_boxplot()+ facet_wrap(~ temperature) + scale_color_manual(values=c("#FFD479", "#9BBB59", "#4F81BD","#C0504D"))+ labs(y="Number of spores", x="Lineages")

#T=25°C: plot

ggplot(data_sp_25_med, aes( lineage,nb_spore, color=lineage))+ geom_boxplot()+ facet_wrap(~ temperature) + scale_color_manual(values=c("#FFD479", "#9BBB59", "#4F81BD","#C0504D"))+ labs(y="Number of spores", x="Lineages")

#T=30°C: plot

ggplot(data_sp_30_med, aes( lineage,nb_spore, color=lineage))+ geom_boxplot()+ facet_wrap(~ temperature) + scale_color_manual(values=c("#FFD479", "#9BBB59", "#4F81BD","#C0504D"))+ labs(y="Number of spores", x="Lineages")

**

#Fit Negative Binomial Generalized Linear Models, and plot residuals

#We chose this type of model because it is suitable for over-dispersed count data.

mod.nb.pois10 <- **glm.nb**(nb_spore**~**lineage, data=data_sp_10_med)

**plotresid**(mod.nb.pois10)

mod.nb.pois15 <- **glm.nb**(nb_spore**~**lineage, data=data_sp_15_med)

**plotresid**(mod.nb.pois15)

mod.nb.pois20 <- **glm.nb**(nb_spore**~**lineage, data=data_sp_20_med)

**plotresid**(mod.nb.pois20)

mod.nb.pois25 <- **glm.nb**(nb_spore**~**lineage, data=data_sp_25_med)

**plotresid**(mod.nb.pois25)

mod.nb.pois30 <- **glm.nb**(nb_spore**~**lineage, data=data_sp_30_med)

**plotresid**(mod.nb.pois30)

#Quantile-quantile plots to check if data are normally distributed

ggqqplot(mod.nb.pois10$residuals)

ggqqplot(mod.nb.pois15$residuals)

ggqqplot(mod.nb.pois20$residuals)

ggqqplot(mod.nb.pois25$residuals)

ggqqplot(mod.nb.pois30$residuals)

#Kruskal-Wallis test (as an alternative to ANOVA, given that data are not normally distributed)

res.kruskal <- data_sp_10_med %>% kruskal_test(nb_spore ~ lineage)

res.kruskal

# A tibble: 1 x 6

.y. n statistic df p method

* <chr> <int> <dbl> <int> <dbl> <chr>

1 nb_spore 39 9.61 3 0.0222 Kruskal-Wallis

res.kruskal <- data_sp_15_med %>% kruskal_test(nb_spore ~ lineage)

res.kruskal

# A tibble: 1 x 6

.y. n statistic df p method

* <chr> <int> <dbl> <int> <dbl> <chr>

1 nb_spore 40 16.8 3 0.000761 Kruskal-Wallis

res.kruskal <- data_sp_20_med %>% kruskal_test(nb_spore ~ lineage)

res.kruskal

# A tibble: 1 x 6

.y. n statistic df p method

* <chr> <int> <dbl> <int> <dbl> <chr>

1 nb_spore 40 6.04 3 0.11 Kruskal-Wallis

res.kruskal <- data_sp_25_med %>% kruskal_test(nb_spore ~ lineage)

res.kruskal

# A tibble: 1 x 6

.y. n statistic df p method

* <chr> <int> <dbl> <int> <dbl> <chr>

1 nb_spore 40 3.42 3 0.331 Kruskal-Wallis

res.kruskal <- data_sp_30_med %>% kruskal_test(nb_spore ~ lineage)

res.kruskal

# A tibble: 1 x 6

.y. n statistic df p method

* <chr> <int> <dbl> <int> <dbl> <chr>

1 nb_spore 41 8.96 3 0.0298 Kruskal-Wallis

#Dunn’s non-parametric multiple comparison test for temperatures for which Kruskal-Wallis tests were significant

PT = dunnTest(nb_spore ~ lineage,  data=data_sp_10_med)

Comparison Z P.unadj P.adj

1 1(Yule) - 2 -2.7164706 0.006598206 0.03958924

2 1(Yule) - 3 -2.2508504 0.024395017 0.12197508

3 2 - 3 0.4439514 0.657077694 0.65707769

4 1(Yule) - 4 -0.7886814 0.430298220 0.86059644

5 2 - 4 1.9073416 0.056476364 0.22590546

6 3 - 4 1.4518571 0.146541356 0.43962407

PT=PT$res

cldList(comparison = PT$Comparison, p.value = PT$P.adj, threshold = 0.05)

Group Letter MonoLetter

1 1(Yule) a a

2 2 b b

3 3 ab ab

4 4 ab ab

PT = dunnTest(nb_spore ~ lineage,  data=data_sp_15_med)

Comparison Z P.unadj P.adj

1 1(Yule) - 2 -2.3573282 0.0184069683 0.073627873

2 1(Yule) - 3 -3.8137339 0.0001368828 0.000821297

3 2 - 3 -1.3206571 0.1866157373 0.559847212

4 1(Yule) - 4 -3.1367872 0.0017081003 0.008540501

5 2 - 4 -0.6769145 0.4984602096 0.996920419

6 3 - 4 0.6613826 0.5083669692 0.508366969

PT=PT$res

cldList(comparison = PT$Comparison, p.value = PT$P.adj, threshold = 0.05)

Group Letter MonoLetter

1 1(Yule) a a

2 2 ab ab

3 3 b b

4 4 b b

PT = dunnTest(nb_spore ~ lineage,  data=data_sp_30_med)

Comparison Z P.unadj P.adj

1 1(Yule) - 2 -0.7036481 0.481651938 1.00000000

2 1(Yule) - 3 -0.5412010 0.588369076 1.00000000

3 2 - 3 0.1587122 0.873895593 0.87389559

4 1(Yule) - 4 -2.8154612 0.004870728 0.02922437

5 2 - 4 -2.0632591 0.039088014 0.15635206

6 3 - 4 -2.2219714 0.026285240 0.13142620

PT=PT$res

cldList(comparison = PT$Comparison, p.value = PT$P.adj, threshold = 0.05)

Group Letter MonoLetter

1 1(Yule) a a

2 2 ab ab

3 3 ab ab

4 4 b b

S7.2. Correlation between number of spores and mycelium size

# normality of data?
shapiro.test(data_spore$myceliumarea) # p-value = 3.4e-12

##
## Shapiro-Wilk normality test
##
## data: data_spore$myceliumarea
## W = 0.95222, p-value = 3.4e-12

ggqqplot(data_spore$myceliumarea, ylab = "mycelium area (cm2)")

shapiro.test(data_spore$spores) # p-value < 2.2e-16

##
## Shapiro-Wilk normality test
##
## data: data_spore$spores
## W = 0.59582, p-value < 2.2e-16

ggqqplot(data_spore$spores, ylab = "number of spores")

# data are not normal
# we can't use Pearson’s test
# => use non parametric tests such as Spearman’s test

# visualize data on scatter plot and test for correlation
ggscatter(data_spore, x = "myceliumarea", y = "spores", title ="All data - Spearman test",
 add = "reg.line", conf.int = TRUE,
 cor.coef = TRUE, cor.method = "spearman",
 xlab = "mycelium area (cm2)", ylab = "number of spores")

ggscatter(data_spore, x = "myceliumarea", y = "spores", title ="", color="lineage",
 add = "reg.line", conf.int = TRUE,
 cor.coef = TRUE, cor.method = "spearman",
 xlab = "mycelium area (cm2)", ylab = "number of spores")

# conclusion: there is a correlation between mycelium area and number of spores when considering all temperatures together

## now per temperature:

data_sp_10 <- subset(data_spore, temperature==10)
data_sp_15 <- subset(data_spore, temperature==15)
data_sp_20 <- subset(data_spore, temperature==20)
data_sp_25 <- subset(data_spore, temperature==25)
data_sp_30 <- subset(data_spore, temperature==30)

###10°C
# normality of the data
shapiro.test(data_sp_10$myceliumarea)

##
## Shapiro-Wilk normality test
##
## data: data_sp_10$myceliumarea
## W = 0.98132, p-value = 0.1588

shapiro.test(data_sp_10$spores)

##
## Shapiro-Wilk normality test
##
## data: data_sp_10$spores
## W = 0.52408, p-value < 2.2e-16

# plot
ggscatter(data_sp_10, x = "myceliumarea", y = "spores", title ="10°C – Spearman’s test",
 add = "reg.line", conf.int = TRUE,
 cor.coef = TRUE, cor.method = "spearman",
 xlab = "mycelium area (cm2) at 10°C", ylab = "number of spores at 10°C")

###15°C
# normality of the data
shapiro.test(data_sp_15$myceliumarea)

##
## Shapiro-Wilk normality test
##
## data: data_sp_15$myceliumarea
## W = 0.98599, p-value = 0.2783

shapiro.test(data_sp_15$spores)

##
## Shapiro-Wilk normality test
##
## data: data_sp_15$spores
## W = 0.70713, p-value = 7.758e-14

# plot
ggscatter(data_sp_15, x = "myceliumarea", y = "spores", title ="15°C – Spearman’s test",
 add = "reg.line", conf.int = TRUE,
 cor.coef = TRUE, cor.method = "spearman",
 xlab = "mycelium area (cm2) at 15°C", ylab = "number of spores at 15°C")

###20°C
# normality of the data
shapiro.test(data_sp_20$myceliumarea)

##
## Shapiro-Wilk normality test
##
## data: data_sp_20$myceliumarea
## W = 0.96086, p-value = 0.004912

shapiro.test(data_sp_20$spores)

##
## Shapiro-Wilk normality test
##
## data: data_sp_20$spores
## W = 0.83435, p-value = 3.718e-09

# plot
ggscatter(data_sp_20, x = "myceliumarea", y = "spores", title ="20°C – Spearman’s test",
 add = "reg.line", conf.int = TRUE,
 cor.coef = TRUE, cor.method = "spearman",
 xlab = "mycelium area (cm2) at 20°C", ylab = "number of spores at 20°C")

###25°C
# normality of the data
shapiro.test(data_sp_25$myceliumarea)

##
## Shapiro-Wilk normality test
##
## data: data_sp_25$myceliumarea
## W = 0.9908, p-value = 0.7997

shapiro.test(data_sp_25$spores)

##
## Shapiro-Wilk normality test
##
## data: data_sp_25$spores
## W = 0.84366, p-value = 3.324e-08

# plot
ggscatter(data_sp_25, x = "myceliumarea", y = "spores", title ="25°C – Spearman’s test",
 add = "reg.line", conf.int = TRUE,
 cor.coef = TRUE, cor.method = "spearman",
 xlab = "mycelium area (cm2) at 25°C", ylab = "number of spores at 25°C")

###30°C
# normality of the data
shapiro.test(data_sp_30$myceliumarea)

##
## Shapiro-Wilk normality test
##
## data: data_sp_30$myceliumarea
## W = 0.9669, p-value = 0.002278

shapiro.test(data_sp_30$spores)

##
## Shapiro-Wilk normality test
##
## data: data_sp_30$spores
## W = 0.58038, p-value < 2.2e-16

# plot
ggscatter(data_sp_30, x = "myceliumarea", y = "spores", title ="30°C – Spearman’s test",
 add = "reg.line", conf.int = TRUE,
 cor.coef = TRUE, cor.method = "spearman",
 xlab = "mycelium area (cm2) at 30°C", ylab = "number of spores at 30°C")

S7.3. Analysis of spore production weighted by mycelium area

####################################
## computing number of spores per cm2
data_spore$spore_per_cm2 <- data_spore$spores/ data_spore$myceliumarea

##medians
data_spore_med_rep <-aggregate(data_spore$spore_per_cm2, data_spore[,c("temperature","lineage","isolate")], FUN=median)
colnames(data_spore_med_rep)[4] <- 'nb_spore'
data_spore_med_rep$nb_spore <- round(data_spore_med_rep$nb_spore*100)

summary(data_spore_med_rep)

## temperature lineage isolate nb_spore
## Min. :10.0 1_international: 0 CH0052 : 5 Min. : 0.0
## 1st Qu.:15.0 1_yule :55 CH0063 : 5 1st Qu.: 6.0
## Median :20.0 2 :48 CH0092 : 5 Median : 74.5
## Mean :20.1 3 :47 CH0110 : 5 Mean : 306.3
## 3rd Qu.:25.0 4 :50 CH0549 : 5 3rd Qu.: 400.0
## Max. :30.0 CH0718 : 5 Max. :2765.0
## (Other):170

####################################
data_sp_10_med <- subset(data_spore_med_rep, temperature==10)
data_sp_15_med <- subset(data_spore_med_rep, temperature==15)
data_sp_20_med <- subset(data_spore_med_rep, temperature==20)
data_sp_25_med <- subset(data_spore_med_rep, temperature==25)
data_sp_30_med <- subset(data_spore_med_rep, temperature==30)

# boxplot number of spores per lineage
ggplot(data_sp_10_med, aes( lineage,nb_spore, color=lineage)) +
 geom_boxplot()+
 facet_wrap(~ temperature) +
 scale_color_manual(values=c("orange","forestgreen","royalblue","firebrick"))+
 labs(y="(Number of spores/ mycelium area)*100", x="Lineage")

ggplot(data_sp_15_med, aes( lineage,nb_spore, color=lineage)) +
 geom_boxplot()+
 facet_wrap(~ temperature) +
 scale_color_manual(values=c("orange","forestgreen","royalblue","firebrick"))+
 labs(y="(Number of spores/ mycelium area)*100", x="Lineage")

ggplot(data_sp_20_med, aes( lineage,nb_spore, color=lineage)) +
 geom_boxplot()+
 facet_wrap(~ temperature) +
 scale_color_manual(values=c("orange","forestgreen","royalblue","firebrick"))+
 labs(y="(Number of spores/ mycelium area)*100", x="Lineage")

ggplot(data_sp_25_med, aes( lineage,nb_spore, color=lineage)) +
 geom_boxplot()+
 facet_wrap(~ temperature) +
 scale_color_manual(values=c("orange","forestgreen","royalblue","firebrick"))+
 labs(y="(Number of spores/ mycelium area)*100", x="Lineage")

ggplot(data_sp_30_med, aes( lineage,nb_spore, color=lineage)) +
 geom_boxplot()+
 facet_wrap(~ temperature) +
 scale_color_manual(values=c("orange","forestgreen","royalblue","firebrick"))+
 labs(y="(Number of spores/ mycelium area)*100", x="Lineage")

##########################################
# non parametric Kruskal-Wallis tests
res.kruskal <- data_sp_10_med %>% kruskal_test(nb_spore ~ lineage) #
res.kruskal$p #p-value =0.0259

## [1] 0.0259

res.kruskal <- data_sp_15_med %>% kruskal_test(nb_spore ~ lineage) #
res.kruskal$p #p-value = 0.000565

## [1] 0.000565

res.kruskal <- data_sp_20_med %>% kruskal_test(nb_spore ~ lineage)
res.kruskal$p #p-value = 0.143

## [1] 0.143

res.kruskal <- data_sp_25_med %>% kruskal_test(nb_spore ~ lineage)
res.kruskal$p #p-value =0.413

## [1] 0.413

res.kruskal <- data_sp_30_med %>% kruskal_test(nb_spore ~ lineage) #
res.kruskal$p #p-value = 0.0223

## [1] 0.0223

#Dunn’s tests for significant Kruskal-Wallis tests

pwc <- data_sp_10_med %>%
 dunn_test(nb_spore ~ lineage, p.adjust.method = "bonferroni")
pwc

## # A tibble: 6 x 9
## .y. group1 group2 n1 n2 statistic p p.adj p.adj.signif
## * <chr> <chr> <chr> <int> <int> <dbl> <dbl> <dbl> <chr>
## 1 nb_spore 1_yule 2 11 9 2.65 0.00800 0.0480 *
## 2 nb_spore 1_yule 3 11 9 2.18 0.0294 0.177 ns
## 3 nb_spore 1_yule 4 11 10 0.692 0.489 1 ns
## 4 nb_spore 2 3 9 9 -0.452 0.651 1 ns
## 5 nb_spore 2 4 9 10 -1.94 0.0529 0.317 ns
## 6 nb_spore 3 4 9 10 -1.47 0.141 0.846 ns

pwc <- data_sp_15_med %>%
 dunn_test(nb_spore ~ lineage, p.adjust.method = "bonferroni")
pwc

## # A tibble: 6 x 9
## .y. group1 group2 n1 n2 statistic p p.adj p.adj.signif
## * <chr> <chr> <chr> <int> <int> <dbl> <dbl> <dbl> <chr>
## 1 nb_spore 1_yule 2 11 9 2.41 0.0159 0.0951 ns
## 2 nb_spore 1_yule 3 11 10 3.93 0.0000863 0.000518 ***
## 3 nb_spore 1_yule 4 11 10 3.11 0.00186 0.0111 *
## 4 nb_spore 2 3 9 10 1.37 0.169 1 ns
## 5 nb_spore 2 4 9 10 0.600 0.549 1 ns
## 6 nb_spore 3 4 10 10 -0.795 0.426 1 ns

pwc <- data_sp_30_med %>%
 dunn_test(nb_spore ~ lineage, p.adjust.method = "bonferroni")
pwc

## # A tibble: 6 x 9
## .y. group1 group2 n1 n2 statistic p p.adj p.adj.signif
## * <chr> <chr> <chr> <int> <int> <dbl> <dbl> <dbl> <chr>
## 1 nb_spore 1_yule 2 11 10 0.836 0.403 1 ns
## 2 nb_spore 1_yule 3 11 10 0.721 0.471 1 ns
## 3 nb_spore 1_yule 4 11 10 2.97 0.00302 0.0181 *
## 4 nb_spore 2 3 10 10 -0.112 0.911 1 ns
## 5 nb_spore 2 4 10 10 2.08 0.0374 0.224 ns
## 6 nb_spore 3 4 10 10 2.19 0.0283 0.170 ns
