# Supplementary material for: Maintenance of divergent lineages of the Rice Blast Fungus Pyricularia oryzae through niche separation, loss of sex and post-mating genetic incompatibilities
Source: PLoS Pathog. 2022 Jul 25;18(7):e1010687. doi: 10.1371/journal.ppat.1010687 (PMC9352207; doi:10.1371/journal.ppat.1010687)
Supplement: S8 Text — (DOCX) [file ppat.1010687.s026.docx]

S8 Text: statistical processing of pathogenicity data.

#R packages (R version 4.0.3)

library(ordinal)

library(RVAideMemoire)

library(lsmeans)

library(FSA)

library(lattice)

library(multcomp)

library(rcompanion)

#import data

patho_data <- read.csv("S13 Data.txt",sep=";",na.strings=c("NA","na"))

patho_data$isolate<- as.factor(patho_data$isolate)

patho_data$clone<- as.factor(patho_data$clone)

patho_data$lineage<- as.factor(patho_data$lineage)

patho_data$ss.lineage<- as.factor(patho_data$ss.lineage)

patho_data$rice_type<- as.factor(patho_data$rice_type)

patho_data$variety<- as.factor(patho_data$variety)

patho_data$notes<-factor(patho_data$symptom_score,levels=c("1","2","3","4","5","6"),ordered=TRUE)

patho_data$typebin<-cut(patho_data$symptom_score,c(0,2,6),include.lowest=TRUE,labels = c("Incompatible","Compatible"))

summary(patho_data)

#Take the median symptom score across replicate

median_score<-aggregate(patho_data$symptom_score, patho_data[,c("isolate","lineage","ss.lineage","rice_type","variety")], FUN=median)

colnames(median_score)[6] <- 'symptom_score'

median_score$symptom_score<-round(median_score$symptom_score,0)

median_score$notes<-factor(median_score$symptom_score,levels=c("1","2","3","4","5","6"),ordered=TRUE)

# fit a proportional odds model

median.clm1<-clm(data=median_score, notes~ss.lineage*rice_type,Hess=TRUE)

median.clm1

## formula: notes ~ ss.lineage * rice_type

## data: median_score

##

## link threshold nobs logLik AIC niter median.grad cond.H

## logit flexible 3080 -4083.68 8245.36 6(2) 6.26e-09 1.4e+04

##

## Coefficients:

## ss.lineage3 ss.lineage4

## 1.130758 0.938238

## ss.lineageBaoshan ss.lineageInt

## 1.053746 0.780031

## ss.lineageLaos ss.lineageYule

## 1.446904 0.998254

## rice_typearomatic rice_typeind

## -0.408516 0.250523

## rice_typejap_temp rice_typejap_trp

## 1.727616 0.006872

## ss.lineage3:rice_typearomatic ss.lineage4:rice_typearomatic

## -0.685654 -0.042992

## ss.lineageBaoshan:rice_typearomatic ss.lineageInt:rice_typearomatic

## -0.062907 -0.309470

## ss.lineageLaos:rice_typearomatic ss.lineageYule:rice_typearomatic

## 0.141134 -0.068622

## ss.lineage3:rice_typeind ss.lineage4:rice_typeind

## 0.093780 -0.397605

## ss.lineageBaoshan:rice_typeind ss.lineageInt:rice_typeind

## -0.053179 -0.106520

## ss.lineageLaos:rice_typeind ss.lineageYule:rice_typeind

## -1.637128 0.801246

## ss.lineage3:rice_typejap_temp ss.lineage4:rice_typejap_temp

## -0.378156 -0.776136

## ss.lineageBaoshan:rice_typejap_temp ss.lineageInt:rice_typejap_temp

## -0.864880 -0.372044

## ss.lineageLaos:rice_typejap_temp ss.lineageYule:rice_typejap_temp

## -1.897844 -0.526060

## ss.lineage3:rice_typejap_trp ss.lineage4:rice_typejap_trp

## 0.262846 -0.089005

## ss.lineageBaoshan:rice_typejap_trp ss.lineageInt:rice_typejap_trp

## 0.145275 0.722665

## ss.lineageLaos:rice_typejap_trp ss.lineageYule:rice_typejap_trp

## -0.541955 0.691068

##

## Threshold coefficients:

## 1|2 2|3 3|4 4|5 5|6

## -1.1428 0.2255 0.4679 0.6320 0.9903

#ANOVA

anova(median.clm1)

## Type I Analysis of Deviance Table with Wald chi-square tests

##

## Df Chisq Pr(>Chisq)

## ss.lineage 6 99.591 < 2.2e-16 ***

## rice_type 4 161.249 < 2.2e-16 ***

## ss.lineage:rice_type 24 97.114 9.288e-11 ***

## ---

## Signif. codes: 0 '***' 0.001 '**' 0.01 '*' 0.05 '.' 0.1 ' ' 1

#Fit a proportional odds model without interactions to carry out posthoc tests

median.clm2<-clm(data=median_score, notes~ss.lineage+rice_type,Hess=TRUE)

median.clm2

## formula: notes ~ ss.lineage + rice_type

## data: median_score

##

## link threshold nobs logLik AIC niter median.grad cond.H

## logit flexible 3080 -4133.45 8296.90 6(2) 7.18e-10 1.1e+03

##

## Coefficients:

## ss.lineage3 ss.lineage4 ss.lineageBaoshan ss.lineageInt

## 0.99189 0.71707 0.91894 0.76240

## ss.lineageLaos ss.lineageYule rice_typearomatic rice_typeind

## 0.75610 1.13995 -0.55516 0.01813

## rice_typejap_temp rice_typejap_trp

## 1.01643 0.15485

##

## Threshold coefficients:

## 1|2 2|3 3|4 4|5 5|6

## -1.26229 0.07864 0.31418 0.47358 0.82169

#ANOVA

anova(median.clm2)

#Contrast lineages using least-squares means

## Type I Analysis of Deviance Table with Wald chi-square tests

##

## Df Chisq Pr(>Chisq)

## ss.lineage 6 103.67 < 2.2e-16 ***

## rice_type 4 163.40 < 2.2e-16 ***

## ---

## Signif. codes: 0 '***' 0.001 '**' 0.01 '*' 0.05 '.' 0.1 ' ' 1

LSM <- lsmeans(median.clm2,~ss.lineage,adjust="sidak")

contrast(LSM,"pairwise")

## contrast estimate SE df z.ratio p.value

## 2 - 3 -0.9919 0.126 Inf -7.867 <.0001

## 2 - 4 -0.7171 0.125 Inf -5.733 <.0001

## 2 - Baoshan -0.9189 0.129 Inf -7.149 <.0001

## 2 - Int -0.7624 0.125 Inf -6.103 <.0001

## 2 - Laos -0.7561 0.122 Inf -6.188 <.0001

## 2 - Yule -1.1399 0.128 Inf -8.911 <.0001

## 3 - 4 0.2748 0.130 Inf 2.106 0.3488

## 3 - Baoshan 0.0730 0.134 Inf 0.546 0.9981

## 3 - Int 0.2295 0.130 Inf 1.760 0.5751

## 3 - Laos 0.2358 0.128 Inf 1.845 0.5170

## 3 - Yule -0.1481 0.133 Inf -1.114 0.9239

## 4 - Baoshan -0.2019 0.133 Inf -1.519 0.7335

## 4 - Int -0.0453 0.130 Inf -0.350 0.9999

## 4 - Laos -0.0390 0.127 Inf -0.307 0.9999

## 4 - Yule -0.4229 0.132 Inf -3.199 0.0233

## Baoshan - Int 0.1565 0.133 Inf 1.179 0.9023

## Baoshan - Laos 0.1628 0.130 Inf 1.250 0.8743

## Baoshan - Yule -0.2210 0.135 Inf -1.634 0.6601

## Int - Laos 0.0063 0.127 Inf 0.050 1.0000

## Int - Yule -0.3775 0.132 Inf -2.859 0.0644

## Laos - Yule -0.3838 0.129 Inf -2.964 0.0478

##

## Results are averaged over the levels of: rice_type

## P value adjustment: tukey method for comparing a family of 7 estimates

cld(LSM)

## ss.lineage lsmean SE df asymp.LCL asymp.UCL .group

## 2 0.0417 0.0851 Inf -0.187 0.27 1

## 4 0.7588 0.0926 Inf 0.510 1.01 2

## Laos 0.7978 0.0889 Inf 0.559 1.04 2

## Int 0.8041 0.0925 Inf 0.556 1.05 23

## Baoshan 0.9606 0.0972 Inf 0.700 1.22 23

## 3 1.0336 0.0940 Inf 0.781 1.29 23

## Yule 1.1816 0.0964 Inf 0.923 1.44 3

##

## Results are averaged over the levels of: rice_type

## Confidence level used: 0.95

## Conf-level adjustment: sidak method for 7 estimates

## P value adjustment: tukey method for comparing a family of 7 estimates

## significance level used: alpha = 0.05

#Contrast rice-types using least-squares means

LSM <- lsmeans(median.clm2,~rice_type,adjust="sidak")

contrast(LSM,"pairwise")

## contrast estimate SE df z.ratio p.value

## aus - aromatic 0.5552 0.102 Inf 5.433 <.0001

## aus - ind -0.0181 0.100 Inf -0.181 0.9998

## aus - jap_temp -1.0164 0.110 Inf -9.245 <.0001

## aus - jap_trp -0.1549 0.103 Inf -1.506 0.5583

## aromatic - ind -0.5733 0.115 Inf -4.966 <.0001

## aromatic - jap_temp -1.5716 0.125 Inf -12.612 <.0001

## aromatic - jap_trp -0.7100 0.118 Inf -6.024 <.0001

## ind - jap_temp -0.9983 0.122 Inf -8.156 <.0001

## ind - jap_trp -0.1367 0.116 Inf -1.178 0.7638

## jap_temp - jap_trp 0.8616 0.125 Inf 6.919 <.0001

##

## Results are averaged over the levels of: ss.lineage

## P value adjustment: tukey method for comparing a family of 5 estimates

cld(LSM)

## rice_type lsmean SE df asymp.LCL asymp.UCL .group

## aromatic 0.115 0.0826 Inf -0.0974 0.327 1

## aus 0.670 0.0603 Inf 0.5152 0.825 2

## ind 0.688 0.0808 Inf 0.4806 0.896 2

## jap_trp 0.825 0.0841 Inf 0.6088 1.041 2

## jap_temp 1.686 0.0934 Inf 1.4466 1.926 3

##

## Results are averaged over the levels of: ss.lineage

## Confidence level used: 0.95

## Conf-level adjustment: sidak method for 5 estimates

## P value adjustment: tukey method for comparing a family of 5 estimates

## significance level used: alpha = 0.05

# generate datasets specific to each rice-type

median_score_aus <- subset(median_score, rice_type == "aus")

median_score_aus <- droplevels(median_score_aus )

median_score_bas <- subset(median_score, rice_type == "aromatic")

median_score_bas <- droplevels(median_score_bas )

median_score_ind <- subset(median_score, rice_type == "ind")

median_score_ind <- droplevels(median_score_ind )

median_score_temp <- subset(median_score, rice_type == "jap_temp")

median_score_temp <- droplevels(median_score_temp )

median_score_trp <- subset(median_score, rice_type == "jap_trp")

median_score_trp <- droplevels(median_score_trp )

#Contrast lineages inoculated onto Aus using least-squares means

median.aus<-clm(data=median_score_aus, notes~ss.lineage,Hess=T)

anova(median.aus)

## Type I Analysis of Deviance Table with Wald chi-square tests

##

## Df Chisq Pr(>Chisq)

## ss.lineage 6 50.585 3.588e-09 ***

## ---

## Signif. codes: 0 '***' 0.001 '**' 0.01 '*' 0.05 '.' 0.1 ' ' 1

LSM <- lsmeans(median.aus,~ss.lineage,adjust="sidak")

contrast(LSM,"pairwise")

## contrast estimate SE df z.ratio p.value

## 2 - 3 -1.1071 0.218 Inf -5.085 <.0001

## 2 - 4 -0.9181 0.218 Inf -4.205 0.0005

## 2 - Baoshan -1.0303 0.222 Inf -4.638 0.0001

## 2 - Int -0.7628 0.216 Inf -3.534 0.0075

## 2 - Laos -1.4213 0.218 Inf -6.532 <.0001

## 2 - Yule -0.9764 0.217 Inf -4.498 0.0001

## 3 - 4 0.1890 0.227 Inf 0.833 0.9815

## 3 - Baoshan 0.0768 0.230 Inf 0.334 0.9999

## 3 - Int 0.3443 0.225 Inf 1.533 0.7251

## 3 - Laos -0.3142 0.225 Inf -1.394 0.8051

## 3 - Yule 0.1307 0.226 Inf 0.579 0.9974

## 4 - Baoshan -0.1122 0.231 Inf -0.486 0.9990

## 4 - Int 0.1553 0.225 Inf 0.689 0.9933

## 4 - Laos -0.5032 0.226 Inf -2.224 0.2827

## 4 - Yule -0.0583 0.227 Inf -0.257 1.0000

## Baoshan - Int 0.2675 0.229 Inf 1.168 0.9061

## Baoshan - Laos -0.3909 0.230 Inf -1.702 0.6146

## Baoshan - Yule 0.0539 0.230 Inf 0.234 1.0000

## Int - Laos -0.6585 0.224 Inf -2.938 0.0516

## Int - Yule -0.2136 0.224 Inf -0.952 0.9639

## Laos - Yule 0.4449 0.225 Inf 1.975 0.4306

##

## P value adjustment: tukey method for comparing a family of 7 estimates

cld(LSM)

## ss.lineage lsmean SE df asymp.LCL asymp.UCL .group

## 2 -0.222 0.146 Inf -0.615 0.170 1

## Int 0.541 0.158 Inf 0.116 0.966 2

## 4 0.696 0.162 Inf 0.262 1.129 2

## Yule 0.754 0.160 Inf 0.325 1.183 2

## Baoshan 0.808 0.167 Inf 0.361 1.255 2

## 3 0.885 0.161 Inf 0.454 1.316 2

## Laos 1.199 0.160 Inf 0.769 1.629 2

##

## Confidence level used: 0.95

## Conf-level adjustment: sidak method for 7 estimates

## P value adjustment: tukey method for comparing a family of 7 estimates

## significance level used: alpha = 0.05

#Contrast lineages inoculated onto Aromatic using least-squares means

median.bas<-clm(data=median_score_bas, notes~ss.lineage,Hess=T)

anova(median.bas)

## Type I Analysis of Deviance Table with Wald chi-square tests

##

## Df Chisq Pr(>Chisq)

## ss.lineage 6 29.585 4.714e-05 ***

## ---

## Signif. codes: 0 '***' 0.001 '**' 0.01 '*' 0.05 '.' 0.1 ' ' 1

LSM <- lsmeans(median.bas,~ss.lineage,adjust="sidak")

contrast(LSM,"pairwise")

## contrast estimate SE df z.ratio p.value

## 2 - 3 -0.4271 0.300 Inf -1.425 0.7884

## 2 - 4 -0.8591 0.306 Inf -2.807 0.0741

## 2 - Baoshan -0.9568 0.308 Inf -3.107 0.0312

## 2 - Int -0.4522 0.303 Inf -1.492 0.7500

## 2 - Laos -1.5316 0.310 Inf -4.934 <.0001

## 2 - Yule -0.8943 0.299 Inf -2.995 0.0437

## 3 - 4 -0.4320 0.309 Inf -1.400 0.8023

## 3 - Baoshan -0.5297 0.310 Inf -1.707 0.6112

## 3 - Int -0.0250 0.307 Inf -0.082 1.0000

## 3 - Laos -1.1044 0.312 Inf -3.538 0.0074

## 3 - Yule -0.4672 0.301 Inf -1.551 0.7139

## 4 - Baoshan -0.0977 0.315 Inf -0.310 0.9999

## 4 - Int 0.4070 0.312 Inf 1.304 0.8503

## 4 - Laos -0.6724 0.316 Inf -2.127 0.3367

## 4 - Yule -0.0352 0.306 Inf -0.115 1.0000

## Baoshan - Int 0.5047 0.314 Inf 1.609 0.6766

## Baoshan - Laos -0.5747 0.317 Inf -1.811 0.5406

## Baoshan - Yule 0.0625 0.308 Inf 0.203 1.0000

## Int - Laos -1.0794 0.316 Inf -3.421 0.0112

## Int - Yule -0.4422 0.305 Inf -1.451 0.7738

## Laos - Yule 0.6372 0.309 Inf 2.062 0.3755

##

## P value adjustment: tukey method for comparing a family of 7 estimates

cld(LSM)

## ss.lineage lsmean SE df asymp.LCL asymp.UCL .group

## 2 -0.624 0.211 Inf -1.190 -0.0582 1

## 3 -0.197 0.215 Inf -0.774 0.3793 12

## Int -0.172 0.220 Inf -0.762 0.4174 12

## 4 0.235 0.222 Inf -0.360 0.8301 123

## Yule 0.270 0.211 Inf -0.297 0.8372 23

## Baoshan 0.332 0.224 Inf -0.267 0.9324 23

## Laos 0.907 0.226 Inf 0.301 1.5136 3

##

## Confidence level used: 0.95

## Conf-level adjustment: sidak method for 7 estimates

## P value adjustment: tukey method for comparing a family of 7 estimates

## significance level used: alpha = 0.05

#Contrast lineages inoculated onto Indica using least-squares means

median.ind<-clm(data=median_score_ind, notes~ss.lineage,Hess=T)

anova(median.ind)

## Type I Analysis of Deviance Table with Wald chi-square tests

##

## Df Chisq Pr(>Chisq)

## ss.lineage 6 52.523 1.463e-09 ***

## ---

## Signif. codes: 0 '***' 0.001 '**' 0.01 '*' 0.05 '.' 0.1 ' ' 1

LSM <- lsmeans(median.ind,~ss.lineage,adjust="sidak")

contrast(LSM,"pairwise")

## contrast estimate SE df z.ratio p.value

## 2 - 3 -1.161 0.299 Inf -3.886 0.0020

## 2 - 4 -0.502 0.287 Inf -1.748 0.5838

## 2 - Baoshan -0.950 0.306 Inf -3.106 0.0313

## 2 - Int -0.635 0.289 Inf -2.196 0.2974

## 2 - Laos 0.164 0.272 Inf 0.604 0.9967

## 2 - Yule -1.717 0.320 Inf -5.360 <.0001

## 3 - 4 0.658 0.306 Inf 2.149 0.3240

## 3 - Baoshan 0.211 0.323 Inf 0.654 0.9949

## 3 - Int 0.526 0.308 Inf 1.710 0.6096

## 3 - Laos 1.325 0.295 Inf 4.497 0.0001

## 3 - Yule -0.556 0.335 Inf -1.658 0.6441

## 4 - Baoshan -0.447 0.314 Inf -1.426 0.7879

## 4 - Int -0.132 0.298 Inf -0.444 0.9994

## 4 - Laos 0.666 0.283 Inf 2.357 0.2170

## 4 - Yule -1.214 0.327 Inf -3.715 0.0038

## Baoshan - Int 0.315 0.315 Inf 1.000 0.9541

## Baoshan - Laos 1.114 0.301 Inf 3.694 0.0041

## Baoshan - Yule -0.767 0.342 Inf -2.241 0.2734

## Int - Laos 0.799 0.284 Inf 2.810 0.0735

## Int - Yule -1.082 0.328 Inf -3.298 0.0169

## Laos - Yule -1.881 0.317 Inf -5.938 <.0001

##

## P value adjustment: tukey method for comparing a family of 7 estimates

cld(LSM)

## ss.lineage lsmean SE df asymp.LCL asymp.UCL .group

## Laos -0.1385 0.189 Inf -0.6445 0.368 1

## 2 0.0256 0.196 Inf -0.5007 0.552 1

## 4 0.5280 0.210 Inf -0.0357 1.092 12

## Int 0.6602 0.212 Inf 0.0909 1.230 12

## Baoshan 0.9752 0.234 Inf 0.3462 1.604 23

## 3 1.1864 0.225 Inf 0.5826 1.790 23

## Yule 1.7424 0.253 Inf 1.0640 2.421 3

##

## Confidence level used: 0.95

## Conf-level adjustment: sidak method for 7 estimates

## P value adjustment: tukey method for comparing a family of 7 estimates

## significance level used: alpha = 0.05

#Contrast lineages inoculated onto Temperate japonica using least-squares means

median.temp<-clm(data=median_score_temp, notes~ss.lineage,Hess=T)

anova(median.temp)

## Type I Analysis of Deviance Table with Wald chi-square tests

##

## Df Chisq Pr(>Chisq)

## ss.lineage 6 17.151 0.008743 **

## ---

## Signif. codes: 0 '***' 0.001 '**' 0.01 '*' 0.05 '.' 0.1 ' ' 1

LSM <- lsmeans(median.temp,~ss.lineage,adjust="sidak")

contrast(LSM,"pairwise")

## contrast estimate SE df z.ratio p.value

## 2 - 3 -0.7789 0.369 Inf -2.112 0.3452

## 2 - 4 -0.1727 0.339 Inf -0.510 0.9987

## 2 - Baoshan -0.2016 0.341 Inf -0.590 0.9971

## 2 - Int -0.4266 0.346 Inf -1.235 0.8807

## 2 - Laos 0.4725 0.313 Inf 1.511 0.7384

## 2 - Yule -0.4916 0.349 Inf -1.407 0.7984

## 3 - 4 0.6062 0.375 Inf 1.615 0.6729

## 3 - Baoshan 0.5773 0.378 Inf 1.527 0.7287

## 3 - Int 0.3523 0.382 Inf 0.923 0.9690

## 3 - Laos 1.2514 0.353 Inf 3.545 0.0072

## 3 - Yule 0.2872 0.385 Inf 0.746 0.9897

## 4 - Baoshan -0.0289 0.349 Inf -0.083 1.0000

## 4 - Int -0.2539 0.353 Inf -0.720 0.9915

## 4 - Laos 0.6452 0.321 Inf 2.009 0.4088

## 4 - Yule -0.3190 0.357 Inf -0.894 0.9735

## Baoshan - Int -0.2250 0.356 Inf -0.633 0.9958

## Baoshan - Laos 0.6741 0.324 Inf 2.079 0.3649

## Baoshan - Yule -0.2900 0.359 Inf -0.807 0.9843

## Int - Laos 0.8991 0.329 Inf 2.737 0.0893

## Int - Yule -0.0650 0.363 Inf -0.179 1.0000

## Laos - Yule -0.9641 0.333 Inf -2.898 0.0577

##

## P value adjustment: tukey method for comparing a family of 7 estimates

cld(LSM)

## ss.lineage lsmean SE df asymp.LCL asymp.UCL .group

## Laos 1.17 0.214 Inf 0.599 1.74 1

## 2 1.64 0.241 Inf 0.998 2.29 12

## 4 1.82 0.253 Inf 1.139 2.50 12

## Baoshan 1.85 0.257 Inf 1.158 2.53 12

## Int 2.07 0.262 Inf 1.367 2.78 12

## Yule 2.14 0.268 Inf 1.417 2.86 12

## 3 2.42 0.293 Inf 1.637 3.21 2

##

## Confidence level used: 0.95

## Conf-level adjustment: sidak method for 7 estimates

## P value adjustment: tukey method for comparing a family of 7 estimates

## significance level used: alpha = 0.05

#Contrast lineages inoculated onto Tropical japonica using least-squares means

median.trp<-clm(data=median_score_trp, notes~ss.lineage,Hess=T)

anova(median.trp)

## Type I Analysis of Deviance Table with Wald chi-square tests

##

## Df Chisq Pr(>Chisq)

## ss.lineage 6 44.117 7.009e-08 ***

## ---

## Signif. codes: 0 '***' 0.001 '**' 0.01 '*' 0.05 '.' 0.1 ' ' 1

LSM <- lsmeans(median.trp,~ss.lineage,adjust="sidak")

contrast(LSM,"pairwise")

## contrast estimate SE df z.ratio p.value

## 2 - 3 -1.5538 0.318 Inf -4.882 <.0001

## 2 - 4 -0.9725 0.310 Inf -3.137 0.0284

## 2 - Baoshan -1.3442 0.322 Inf -4.171 0.0006

## 2 - Int -1.6580 0.323 Inf -5.134 <.0001

## 2 - Laos -1.0223 0.309 Inf -3.311 0.0162

## 2 - Yule -1.8528 0.334 Inf -5.548 <.0001

## 3 - 4 0.5813 0.317 Inf 1.831 0.5268

## 3 - Baoshan 0.2095 0.328 Inf 0.639 0.9955

## 3 - Int -0.1043 0.328 Inf -0.318 0.9999

## 3 - Laos 0.5315 0.316 Inf 1.680 0.6294

## 3 - Yule -0.2990 0.338 Inf -0.884 0.9751

## 4 - Baoshan -0.3718 0.322 Inf -1.154 0.9111

## 4 - Int -0.6856 0.322 Inf -2.126 0.3372

## 4 - Laos -0.0498 0.310 Inf -0.161 1.0000

## 4 - Yule -0.8803 0.333 Inf -2.643 0.1132

## Baoshan - Int -0.3138 0.333 Inf -0.942 0.9657

## Baoshan - Laos 0.3220 0.321 Inf 1.003 0.9536

## Baoshan - Yule -0.5086 0.343 Inf -1.482 0.7557

## Int - Laos 0.6358 0.321 Inf 1.978 0.4286

## Int - Yule -0.1947 0.343 Inf -0.567 0.9977

## Laos - Yule -0.8305 0.332 Inf -2.502 0.1583

##

## P value adjustment: tukey method for comparing a family of 7 estimates

cld(LSM)

## ss.lineage lsmean SE df asymp.LCL asymp.UCL .group

## 2 -0.284 0.215 Inf -0.8609 0.293 1

## 4 0.689 0.221 Inf 0.0946 1.283 2

## Laos 0.739 0.220 Inf 0.1486 1.328 2

## Baoshan 1.060 0.238 Inf 0.4225 1.699 2

## 3 1.270 0.232 Inf 0.6478 1.892 2

## Int 1.374 0.239 Inf 0.7343 2.014 2

## Yule 1.569 0.253 Inf 0.8901 2.248 2

##

## Confidence level used: 0.95

## Conf-level adjustment: sidak method for 7 estimates

## P value adjustment: tukey method for comparing a family of 7 estimates

## significance level used: alpha = 0.05
