# Supplementary material for: Maintenance of divergent lineages of the Rice Blast Fungus Pyricularia oryzae through niche separation, loss of sex and post-mating genetic incompatibilities
Source: PLoS Pathog. 2022 Jul 25;18(7):e1010687. doi: 10.1371/journal.ppat.1010687 (PMC9352207; doi:10.1371/journal.ppat.1010687)
Supplement: S1 Fig — (DOCX) [file ppat.1010687.s032.docx]

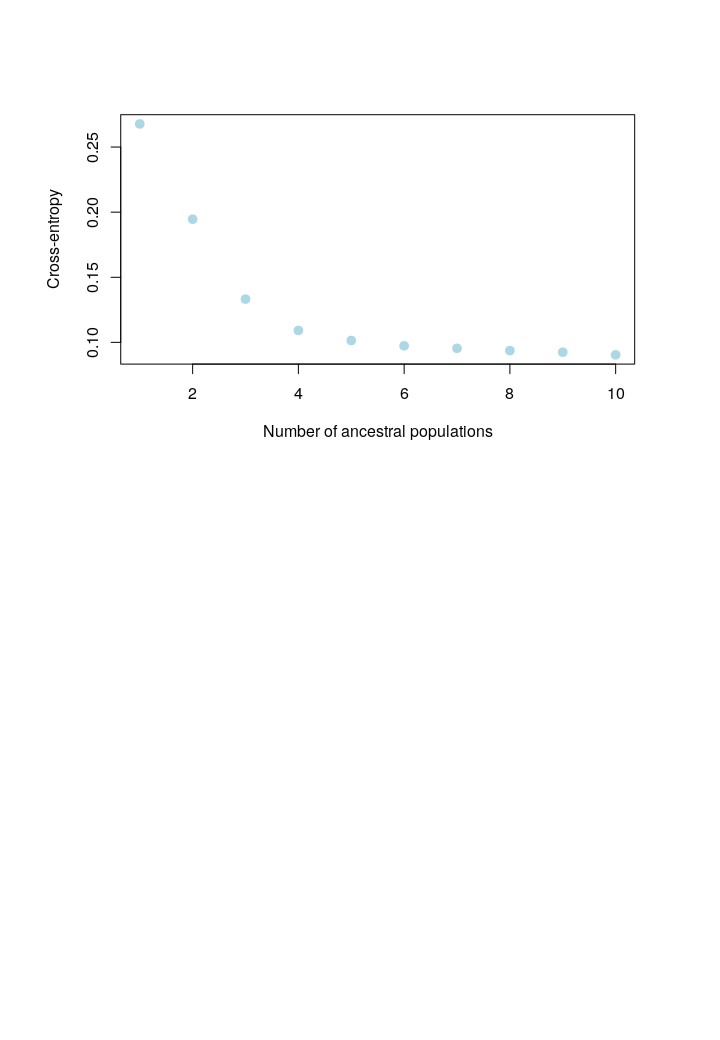


S1 Fig. Cross-entropy (CE) as a function of the number of clusters K modeled in sNMF analyses of population subdivision.
