# Supplementary material for: Maintenance of divergent lineages of the Rice Blast Fungus Pyricularia oryzae through niche separation, loss of sex and post-mating genetic incompatibilities
Source: PLoS Pathog. 2022 Jul 25;18(7):e1010687. doi: 10.1371/journal.ppat.1010687 (PMC9352207; doi:10.1371/journal.ppat.1010687)
Supplement: S3 Fig — (DOCX) [file ppat.1010687.s034.docx]

(A)

(B)

S3 Fig. Linkage disequilibrium (*r^2^*) as a function of physical distance (kb) in four lineages of P. oryzae (A) and in clusters within lineage 1 (B).
