# Supplementary material for: Maintenance of divergent lineages of the Rice Blast Fungus Pyricularia oryzae through niche separation, loss of sex and post-mating genetic incompatibilities
Source: PLoS Pathog. 2022 Jul 25;18(7):e1010687. doi: 10.1371/journal.ppat.1010687 (PMC9352207; doi:10.1371/journal.ppat.1010687)
Supplement: S4 Fig — For each lineage, genomes were resampled in < = 2000 combinations of N-1 genomes (N being the sample size). (A) Non-effector genes. (B) Putative effectors. (DOCX) [file ppat.1010687.s035.docx]

1. Non-effector genes


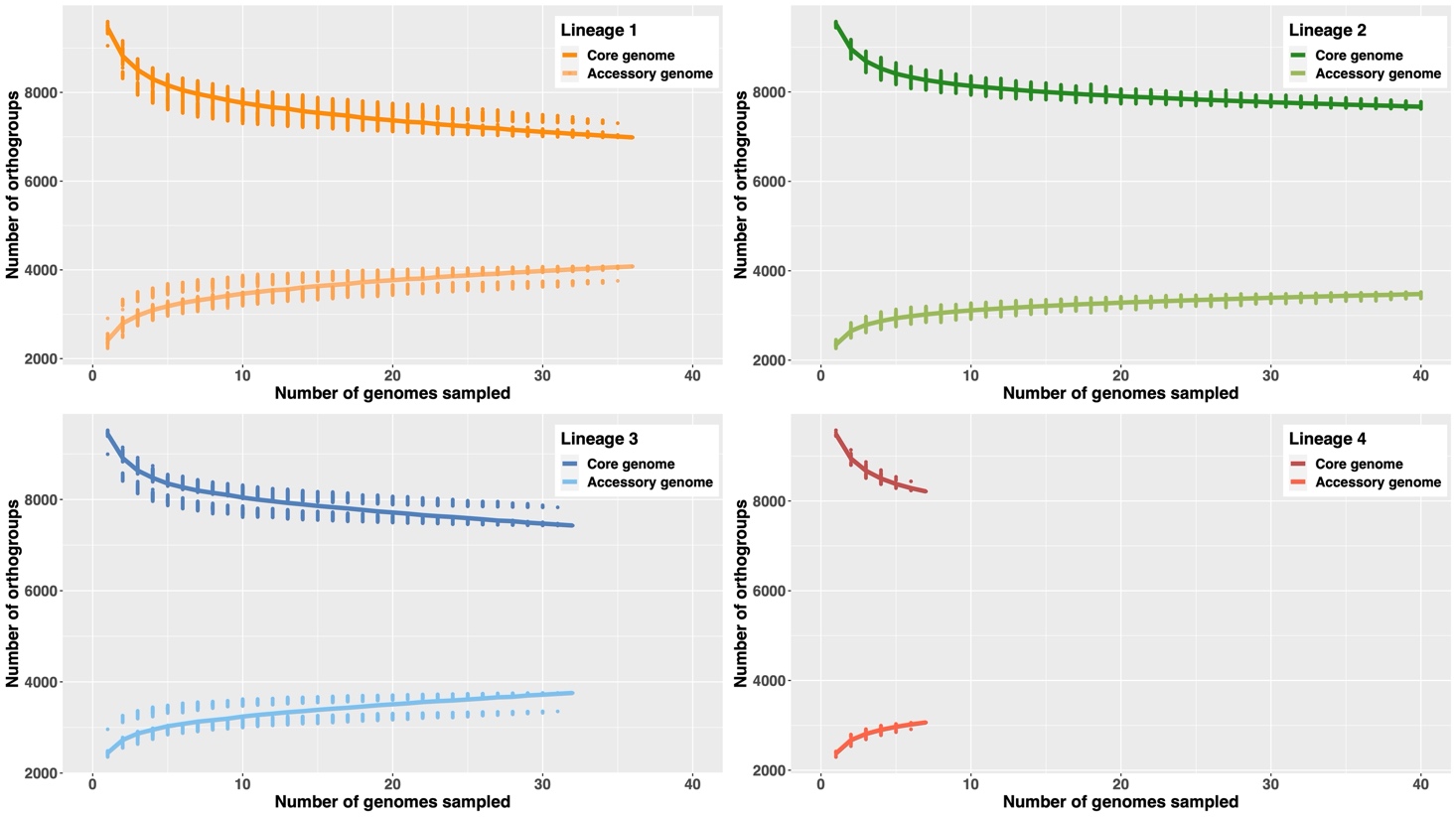


1. Putative effectors


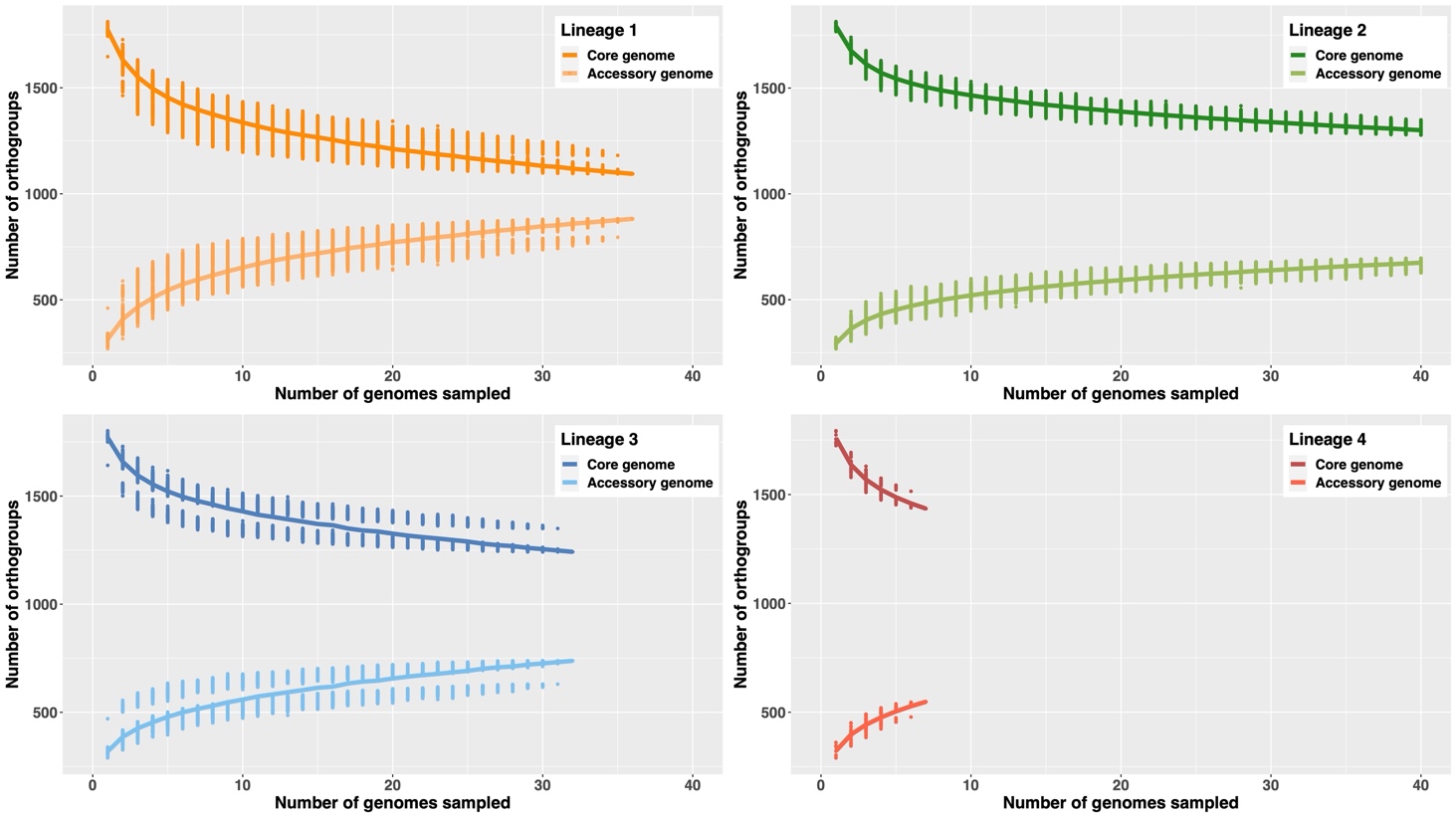


S4 Fig. Estimating the size of the core and accessory genome using a rarefaction approach. For each lineage, genomes were resampled in <=2000 combinations of N-1 genomes (N being the sample size). (A) Non-effector genes. (B) Putative effectors.
