# Supplementary material for: Maintenance of divergent lineages of the Rice Blast Fungus Pyricularia oryzae through niche separation, loss of sex and post-mating genetic incompatibilities
Source: PLoS Pathog. 2022 Jul 25;18(7):e1010687. doi: 10.1371/journal.ppat.1010687 (PMC9352207; doi:10.1371/journal.ppat.1010687)
Supplement: S5 Fig — Size of core and accessory genomes for non-effector proteins (A) and putative effectors (B) estimated using a rarefaction approach with a pseudo-sample size of n = 30 genomes. Lineage 4 was not included due to small sample size. (DOCX) [file ppat.1010687.s036.docx]

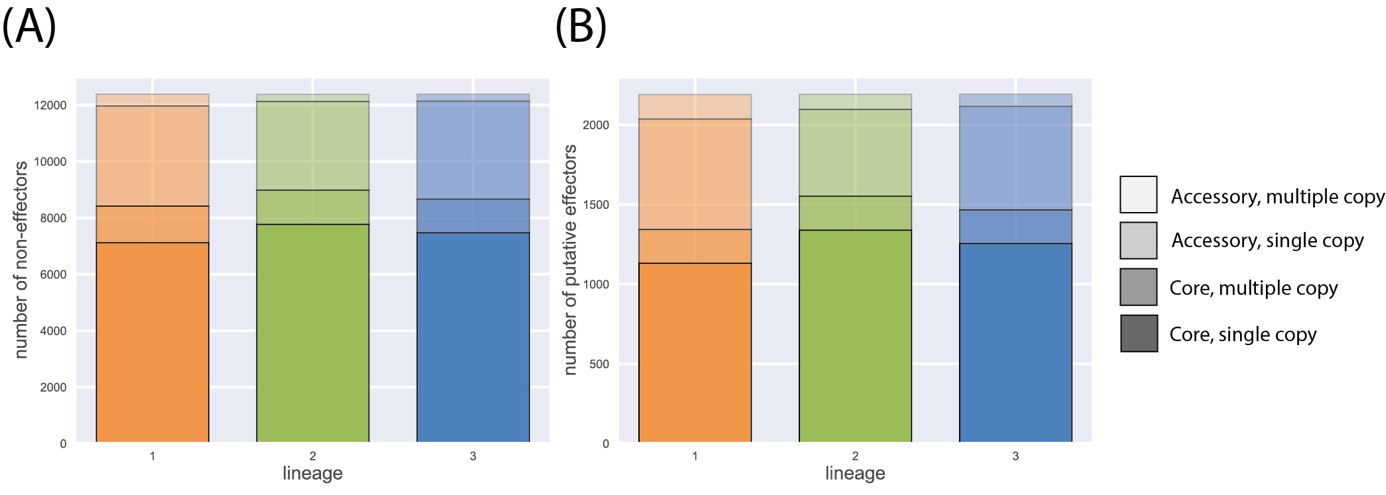


S5 Fig. Size of core and accessory genomes for non-effector proteins (A) and putative effectors (B) estimated using a rarefaction approach with a pseudo-sample size of n=30 genomes. Lineage 4 was not included due to small sample size.
