# Supplementary material for: Maintenance of divergent lineages of the Rice Blast Fungus Pyricularia oryzae through niche separation, loss of sex and post-mating genetic incompatibilities
Source: PLoS Pathog. 2022 Jul 25;18(7):e1010687. doi: 10.1371/journal.ppat.1010687 (PMC9352207; doi:10.1371/journal.ppat.1010687)
Supplement: S6 Fig — The x-axis represents the outlying mean index (OMI), which measures the distance between the mean habitat conditions used by a lineage and the mean habitat conditions used by the entire species, to test the hypothesis that different lineages are distributed in regions with different climates. (DOCX) [file ppat.1010687.s037.docx]

S6 Fig: ecological niches of the four major lineages (referred to as L1 to L4) considering each of the 19 biomes individually (referred to as Bio1 to Bio19). The x-axis represents the outlying mean index (OMI), which measures the distance between the mean habitat conditions used by a lineage and the mean habitat conditions used by the entire species, to test the hypothesis that different lineages are distributed in regions with different climates. The red vertical line represents the mean value for the entire species. Black dots represent the niche position of lineages and horizontal bars represent niche breadth, along the first axis of the OMI. The lineages order is different for the different biomes because lineages are ordered along the y-axis by increasing niche position.
